# Association of body mass index with amnestic and non-amnestic mild cognitive impairment risk in elderly

**DOI:** 10.1186/s12888-017-1493-x

**Published:** 2017-09-15

**Authors:** Feng Wang, Minghui Zhao, Zhaoli Han, Dai Li, Shishuang Zhang, Yongqiang Zhang, Xiaodong Kong, Ning Sun, Qiang Zhang, Ping Lei

**Affiliations:** Department of Geriatrics, Tianjin Medical University General Hospital; Tianjin Geriatrics Institute, No. 154, Anshan Road, Heping District, Tianjin, 300052 China

**Keywords:** Body mass index, Dementia, Mild cognitive impairment, Obesity, Weight

## Abstract

**Background:**

Previous studies focused on the relationship between body mass index and cognitive disorder and obtained many conflicting results. This study explored the potential effects of body mass index on the risk of mild cognitive impairment (amnestic and non-amnestic) in the elderly.

**Methods:**

The study enrolled 240 amnestic mild cognitive impairment patients, 240 non-amnestic mild cognitive impairment patients and 480 normal cognitive function controls. Data on admission and retrospective data at baseline (6 years ago) were collected from their medical records. Cognitive function was evaluated using Mini-Mental State Examination and Montreal Cognitive Assessment.

**Results:**

Being underweight, overweight or obese at baseline was associated with an increased risk of amnestic mild cognitive impairment (*OR*: 2.30, 95%*CI*: 1.50 ~ 3.52; *OR*: 1.74, 95%*CI*: 1.36 ~ 2.20; *OR*: 1.71, 95%*CI*: 1.32 ~ 2.22, respectively). Being overweight or obese at baseline was also associated with an increased risk of non-amnestic mild cognitive impairment (*OR*: 1.51, 95%*CI*: 1.20 ~ 1.92; *OR*: 1.52, 95%*CI*: 1.21 ~ 1.97, respectively). In subjects with normal weights at baseline, an increased or decreased body mass index at follow-up was associated with an elevated risk of amnestic mild cognitive impairment (*OR*: 1.80, 95%*CI*: 1.10 ~ 3.05; *OR*: 3.96, 95%*CI*: 2.88 ~ 5.49, respectively), but only an increased body mass index was associated with an elevated risk of non-amnestic mild cognitive impairment (*OR*: 1.71, 95%*CI*: 1.16 ~ 2.59).

**Conclusions:**

Unhealthy body mass index levels at baseline and follow-up might impact the risk of both types of mild cognitive impairment (amnestic and non-amnestic).

## Background

Cognitive disorder is a common nervous system disease in the elderly, and it includes mild cognitive impairment (MCI), Alzheimer’s disease (AD) and vascular dementia (VD) [1]. In recent decades, cognitive disorder has become one of the most important geriatric health problems. Around the world, dementia affected more than 45 million people in 2015 [2]. Many patients with dementia have a poor quality of life with some loss of dignity [3]. Individuals with MCI have a higher risk of dementia [4]. Some interventions are therefore needed to terminate or slow the progression of MCI [5].

An increasing number of studies have focused on the relationship between body mass index (BMI) and cognitive disorder in elderly people. Ye et al. revealed that being underweight at baseline was associated with a higher risk of progression to AD, whereas obesity at baseline predicted a lower risk [6]. Furthermore, a significant increase or decrease in BMI during the follow-up period reflected an increased risk of progression to AD [6]. Horie et al. reported that intentional weight loss through diet was associated with cognitive improvement in obese patients with MCI [7]. However, Alhurani et al. reported that increasing weight loss per decade from midlife to late life was a marker for MCI and might help identify persons who are at an increased risk for MCI [8]. This topic has not been well investigated, and there are many conflicting results.

MCI can be divided into amnestic MCI (aMCI) and non-amnestic MCI (naMCI). Memory loss is the major symptom of aMCI, which has a higher risk to convert to AD [9]. The non-amnestic form of MCI includes deficits other than memory and constitutes a higher risk to convert to other dementia forms such as diffuse Lewy body dementia [10]. Therefore, these two types of MCI may be implicated in diverse pathogenesis of dementia.

The associations of BMI with aMCI and naMCI may also be different, but this inference has not been proved. Therefore, we conducted a retrospective observational study enrolling nearly 1000 subjects to clarify the potential effects of baseline disorder and the follow-up changes of BMI on the risk of aMCI and naMCI in elderly people.

## Methods

### Subjects

Tianjin Medical University General Hospital Geriatrics Department provides medical services to the elderly in the district. Elderly individuals attend the Geriatric Outpatient Department of this hospital for health examination and disease counselling every 1 to 2 years. After permission, health data of the elderly have been collected for further research since 2008.

In the study, the inclusion criteria for MCI patients were predefined as follows: (1) Outpatients in Geriatrics Department, Tianjin Medical University General Hospital between January 1, 2014, and October 31, 2016, (2) over 60 years of age on admission, (3) meet the diagnostic criteria of aMCI or naMCI. (4) have medical records from the past 6 years, (5) have no history of MCI during the previous 6 years, and (6) agree to participate in the study. Overall, 240 patients with aMCI and 240 patients with naMCI were consecutively enrolled in the study according to the inclusion criteria.

A total of 480 controls with normal cognitive function were randomly selected from the Medical Examination Department, Tianjin Medical University General Hospital in the same period. All controls were over 60 years of age on admission.

Subjects who had Alzheimer’s disease, vascular dementia, other types of dementia, myocardial infarction, cerebral infarction, hematencephalon, malignancies and mental diseases were excluded from the study.

All subjects or their legal guardians signed written informed consent forms. The study was approved by the Ethics Committee of Tianjin Medical University General Hospital.

### Data collection

This was a retrospective observational study. Data on admission and at baseline were collected from medical records. “On admission” was defined as “January 1, 2014 and October 31, 2016”, and “at baseline” was defined as “approximately 6 years ago, between January 1, 2008 and October 31, 2010”. The follow-up period was 6 years for each MCI patient. The data collected from the medical records included demographic data, height, weight, education level, medical history, medication history, cognitive function and other useful information. In addition, genotyping was conducted in the subjects to determine their apolopoprotein E4 (APOE4) carrier status on admission.

### Diagnosis of mild cognitive impairment

In the Geriatrics Department and Medical Examination Department, Mini-Mental State Examination (MMSE) and Montreal Cognitive Assessment (MoCA) were adopted for preliminary cognitive function determination. A subject with suspected cognitive disorders was recommended to a psychologist for further neurologic examination. The MCI patients were determined according to the diagnostic criteria (from Petersens et al.) by the psychologist [11]. The diagnosis was also approved by another psychologist.

The diagnostic criteria of MCI were as follows: (1) Evidence of memory or other cognitive disorders, (2) preservation of general cognitive and functional abilities, and (3) absence of diagnosed dementia. MCI patients with and without memory disorder were diagnosed separately with aMCI and naMCI.

Cognitive function was evaluated using MMSE and MoCA. In MMSE, subjects with fewer than 20 points and 24 points were considered to have cognitive disorder in subjects with primary school education and more than primary school education, respectively. In MoCA, subjects with fewer than 25 points and 26 points were considered to have cognitive disorder in subjects with equal to or less than 12 years of education and more than 12 years of education, respectively [12, 13].

A subject without suspected cognitive disorders or with normal scores in MMSE and MoCA was considered a subject with normal cognitive function.

### Body mass index and weight

BMI was calculated using a formula: BMI (kg/m^2^) = weight (kg) / height ^2^ (m^2^). Underweight (BMI <18.5 kg/m^2^), normal weight (18.5 ~ 22.9 kg/m^2^), overweight (23.0 ~ 24.9 kg/m^2^) and obesity (BMI ≥25 kg/m^2^) were defined according to the World Health Organization’s recommendations for Asian populations [14]. The subjects with an increased BMI (BMI increase of >4% per year), decreased BMI (decrease of >4% per year) or stable BMI (increase or decrease of ≤4% per year) were categorized according to the 4% cut-off for BMI [15].

### Statistical analysis

Continuous and categorical variables were shown by mean ± standard deviation (SD) and frequency. The difference of multiple continuous variables was determined using one-way variance analysis (ANOVA) with Duncan’s post hoc test. The difference of categorical variables was determined using chi-square test. If a *P* value was less than 0.05, it was considered statistical significance. The association of MCI risk with BMI was evaluated using multivariate logistic regression analysis. The odds ratio (*OR*) and 95% confidence interval (*CI*) were calculated. If a 95%*CI* did not include the value “1”, it was considered statistically significant. All analyses were conducted using SPSS version 19.0 (SPSS, iNC., Chicago, IL, USA).

## Results

As shown in Table 1, there were more subjects with APOE4, type 2 diabetes mellitus (DM), hypertension, coronary heart disease (CHD) and metabolism syndrome (MS) in the aMCI and naMCI groups than in the control group at baseline (*P* < 0.05). More subjects had sulfonylureas, metformin and statins in the aMCI and naMCI groups compared with the control group (*P* < 0.05). Furthermore, the subjects in the naMCI group were more likely to have hypertension and CHD than the subjects in the aMCI group at baseline (*P* < 0.05).

**Table 1:** Baseline characteristics of patients with mild cognitive impairment and controls with normal cognitive function in the study

Baseline	aMCI ^a^ (n = 240)	naMCI ^a^ (*n* = 240)	Control (*n* = 480)
Male (n, %)	170 (70.8)	176 (73.3)	360 (75.0)
Age (yrs, mean ± SD) ^a^	73.4 ± 7.7	72.6 ± 9.6	73.2 ± 6.8
Han nationality (n, %)	224 (93.3)	220 (91.7)	460 (95.8)
Education			
≤ 12 years	112 (46.7)	107 (44.6)	231 (48.1)
>12 years	128 (53.3)	133 (55.4)	249 (51.9)
Type 2 DM (n, %) ^a^	70 (29.2) *	66 (27.5) *	84 (17.5)
Hypertension (n, %)	170 (70.8) * ^#^	198 (82.5) *	256 (53.3)
CHD (n, %) ^a^	98 (40.8) * ^#^	134 (55.8) *	146 (30.4)
MS (n, %) ^a^	62 (25.8) *	60 (25.0) *	76 (15.8)
Sulfonylureas (n, %)	33 (13.8) *	35 (14.6) *	41 (8.5)
Metformin (n, %)	45 (18.8) *	43 (17.9) *	61 (12.7)
Insulin (n, %)	13 (5.4)	10 (4.2)	15 (3.1)
Statins (n, %)	53 (22.1) *	58 (24.2) *	70 (14.6)
APOE4 carriers (n, %) ^a^	72 (30.0) *	61 (25.4) *	90 (18.8)

^a^ *SD* standard deviation, *DM* diabetes mellitus, *CHD* coronary heart disease, *MS* metabolism syndrome, *APOE4* apolopoprotein E4, *aMCI* amnestic mild cognitive impairment, *naMCI* non-amnestic mild cognitive impairment

* Compared with control, *P* < 0.05; ^#^ Compared with naMCI, *P* < 0.05

As shown in Table 2, there was no significant difference in the scores of MMSE and MoCA among the aMCI, naMCI and control groups at baseline (*P* > 0.05), which indicated that the subjects in the aMCI and naMCI groups had normal cognitive function at baseline. On admission, the scores of MMSE and MoCA were markedly decreased in the aMCI and naMCI groups than in the control group (*P* < 0.001), indicating that the subjects showed obvious cognitive disorders after 6 years.

**Table 2:** Scores of neuropsychologic examination in patients with mild cognitive impairment and the controls with normal cognitive function in the study

	aMCI ^a^ (n = 240)	naMCI ^a^ (n = 240)	Control (n = 480)	*P* value ^c^
MMSE (mean ± SD) ^a^				
Total at baseline	28.0 ± 1.1	28.1 ± 1.1	28.0 ± 1.1	0.313
Underweight at baseline	27.7 ± 1.3	27.2 ± 0.7	27.9 ± 1.0	0.640
Normal weight at baseline	28.0 ± 1.2	28.1 ± 1.2	28.0 ± 1.1	0.765
Overweight at baseline	27.9 ± 1.0	28.4 ± 1.1	28.2 ± 1.2	0.080
Obese at baseline	27.9 ± 1.1	27.9 ± 1.1	28.0 ± 1.1	0.881
Total on admission	24.1 ± 1.2 *	24.1 ± 1.1 *	28.1 ± 1.2	<0.001
*P* value ^b^	<0.001	<0.001	0.672	
MoCA (mean ± SD) ^a^				
Total at baseline	28.4 ± 1.1	28.5 ± 1.1	28.4 ± 1.1	0.529
Underweight at baseline	28.6 ± 1.3	28.7 ± 1.4	28.4 ± 1.0	0.872
Normal weight at baseline	28.4 ± 1.1	28.5 ± 1.1	28.4 ± 1.1	0.684
Overweight at baseline	28.3 ± 1.1	28.4 ± 1.2	28.4 ± 1.2	0.835
Obese at baseline	28.4 ± 1.1	28.6 ± 1.1	28.4 ± 1.1	0.481
Total on admission	24.4 ± 1.1 *	24.4 ± 1.1 *	28.4 ± 1.1	<0.001
P value ^b^	<0.001	<0.001	0.690	

^a^ *MMSE* Mini-Mental State Examination, *MoCA* Montreal Cognitive Assessment, *SD* standard deviation, *aMCI* amnestic mild cognitive impairment, *naMCI* non-amnestic mild cognitive impairment

^b^ Difference of the scores between total at baseline and total on admission was determined by independent sample t test. If a *P* value < 0.05, it was considered significant

^c^ Difference of the scores among aMCI, naMCI and control groups was determined by one-way variance analysis (ANOVA) with Duncan’s post hoc test. If a *P* value < 0.05, it was considered significant. * Compared with control, *P* < 0.05

As shown in Table 3, there was no difference in the baseline level of BMI among the aMCI, naMCI and control groups (*P* > 0.05), and there was no difference in the admission level (after 6 years) of BMI among these three groups (*P* > 0.05). The subjects were defined as underweight, normal weight, overweight or obese subjects according to their baseline BMI values. The aMCI and naMCI groups had more overweight and obese subjects (*P* < 0.05), and the control group had more normal weight subjects (*P* < 0.05). The underweight subjects were more common in the aMCI group than in the naMCI and control groups (*P* < 0.05). The subjects were also defined as increased BMI, stable BMI and decreased BMI subjects according to the change in BMI from baseline to admission (approximately 6 years). The increased BMI, stable BMI and decreased BMI subjects were equally distributed among the aMCI, naMCI and control groups (*P* > 0.05).

**Table 3:** Body mass index of patients with mild cognitive impairment and the controls with normal cognitive function in the study

	aMCI ^a^ (n = 240)	naMCI ^a^ (*n* = 240)	Control (*n* = 480)
BMI at baseline (kg/m^2^, mean ± SD) ^a, b^	23.5 ± 4.0	23.3 ± 3.1	23.5 ± 4.1
BMI on admission (kg/m^2^, mean ± SD)	23.6 ± 3.3	23.4 ± 3.0	23.5 ± 2.9
BMI at baseline			
Underweight (n, %)	11 (4.6) * ^#^	4 (1.7)	9 (1.9)
Normal weight (n, %)	87 (36.3) *	100 (41.7) *	267 (55.6)
Overweight (n, %)	77 (32.1) *	73 (30.4) *	110 (22.9)
Obese (n, %)	65 (27.1) *	63 (26.3) *	94 (19.6)
BMI change			
Increased (n, %)	33 (13.8)	28 (11.7)	52 (10.4)
Stable (n, %)	181 (75.4)	188 (78.3)	392 (81.7)
Decreased (n, %)	26 (10.8)	24 (10.0)	38 (7.9)

^a^ *BMI* body mass index, *SD* standard deviation, *aMCI* amnestic mild cognitive impairment, *naMCI* non-amnestic mild cognitive impairment

^b^ Difference of three continuous variables was determined by one-way variance analysis (ANOVA) with Duncan’s post hoc test. Difference of categorical variables was determined using chi-square test. If a *P* value < 0.05, it was considered significant. * Compared with control, *P* < 0.05; # Compared with naMCI, *P* < 0.05

As shown in Table 4, being underweight, overweight or obese at baseline was associated with an increased risk of aMCI in the aMCI group (*OR*: 2.30, 95%*CI*: 1.50 ~ 3.52; *OR*: 1.74, 95%*CI*: 1.36 ~ 2.20; *OR*: 1.71, 95%*CI*: 1.32 ~ 2.22, respectively). In the naMCI group, being overweight or obese but not underweight at baseline was associated with an increased risk of naMCI (*OR*: 1.51, 95%*CI*: 1.20 ~ 1.92; *OR*: 1.52, 95%*CI*: 1.21 ~ 1.97; *OR*: 1.19, 95%*CI*: 0.55 ~ 2.66, respectively). After 6 years, subjects with an increased or decreased BMI did not exhibit any changes in MCI risk in the aMCI or naMCI groups.

**Table 4:** Association of body mass index with amnestic and non-amnestic mild cognitive impairment risk

	aMCI (n)	Control (n)	aMCI vs Control OR (95%CI) ^a, b^	naMCI (n)	Control (n)	naMCI vs Control OR (95%CI) ^a, b^
BMI at baseline ^a^						
Normal weight	87	267	Reference	100	267	Reference
Underweight	11	9	2.30 (1.50 ~ 3.52)	4	9	1.19 (0.55 ~ 2.66)
Overweight	77	110	1.74 (1.36 ~ 2.20)	73	110	1.51 (1.20 ~ 1.92)
Obese	65	94	1.71 (1.32 ~ 2.22)	63	94	1.52 (1.21 ~ 1.97)
BMI change						
Stable	181	392	Reference	188	392	Reference
Increased	33	52	1.27 (0.97 ~ 1.70)	28	52	1.11 (0.81 ~ 1.55)
Decreased	26	38	1.35 (0.99 ~ 1.85)	24	38	1.23 (0.89 ~ 1.72)

^a^ *BMI* body mass index, *aMCI* amnestic mild cognitive impairment, *naMCI* non-amnestic mild cognitive impairment, *OR* odds ratio, *CI* confidence interval

^b^ Multivariable logistic regression analysis was adjusted by gender, age, race, education level, medication history, apolopoprotein E4 carrier, onset of type 2 diabetes mellitus, hypertension, coronary heart disease and metabolism syndrome

As shown in Table 5, increased or decreased BMI in subjects with a normal weight at baseline were associated with an elevated risk of aMCI (*OR*: 1.80, 95%*CI*: 1.10 ~ 3.05; *OR*: 3.96, 95%*CI*: 2.88 ~ 5.49, respectively). However, only an increased BMI in the subjects with a normal weight at baseline was associated with an elevated risk of naMCI (*OR*: 1.71, 95%*CI*: 1.16 ~ 2.59). In addition, a decreased BMI in the subjects who were overweight or obese at baseline was related to a decreased risk of aMCI in the study (*OR*: 0.19, 95%*CI*: 0.09 ~ 0.63).

**Table 5:** Association of body mass index change with amnestic and non-amnestic mild cognitive impairment risk according to baseline body mass index level

	aMCI (n)	Control (n)	aMCI vs Control OR (95%CI) ^a, b^	naMCI (n)	Control (n)	naMCI vs Control OR (95%CI) ^a, b^
Underweight at baseline						
Stable BMI ^a^	10	4	Reference	3	4	Reference
Increased BMI	1	5	0.27 (0.09 ~ 1.51)	1	5	0.43 (0.09 ~ 2.86)
Normal weight at baseline						
Stable BMI	51	231	Reference	73	222	Reference
Increased BMI	12	26	1.80 (1.10 ~ 3.05)	17	24	1.71 (1.16 ~ 2.59)
Decreased BMI	24	10	3.96 (2.88 ~ 5.49)	10	21	1.35 (0.79 ~ 2.27)
Overweight or obese at baseline						
Stable BMI	120	155	Reference	112	162	Reference
Increased BMI	20	21	1.17 (0.85 ~ 1.62)	10	25	0.81 (0.52 ~ 1.41)
Decreased BMI	2	28	0.19 (0.09 ~ 0.63)	14	17	1.17 (0.80 ~ 1.78)

^a^ *BMI* body mass index, *aMCI* amnestic mild cognitive impairment, *naMCI* non-amnestic mild cognitive impairment, *OR* odds ratio, *CI* confidence interval

^b^ Multivariable logistic regression analysis was adjusted by gender, age, race, education level, medication history, apolopoprotein E4 carrier, onset of type 2 diabetes mellitus, hypertension, coronary heart disease and metabolism syndrome

## Discussion

In this study, several of the recognized risk factors for MCI were not equivalent among the aMCI, naMCI and control groups. For example, onset of type 2 DM, hypertension and CHD at baseline were more common in the aMCI and naMCI groups compared with the control group. These findings were consistent with previous studies [16–18]. Two vascular-related diseases (hypertension and CHD) were more dominant in the naMCI group than in the aMCI group. This result partly proved that naMCI might be more closely related to vascular disorders, and was also consistent with some previous studies [19–21]. In addition, some hypoglycemic and hypolipidemic therapies, such as sulfonylureas, metformin, insulin and statins, affected the body weight and BMI [22]. In the study, these factors were also not distributed equally among the three groups.

Considering the confounding factors mentioned above, multivariate logistic regression analysis was adopted in the study. It revealed that being overweight or obese at baseline was associated with an approximately 50-70% increased risk of all types of MCI (aMCI and naMCI). The possible explanation is that obesity triggered both vascular and non-vascular disorders (e.g. atherosclerosis and inflammation) and promoted the risk of cognitive impairment [23, 24]. However, being underweight at baseline was related only to an increased risk of aMCI but not of naMCI. There were two possible explanations. First, the sample size was too small, and the power of the test was limited. Second, being underweight might keep people away from many vascular risk factors and reduce the risk of naMCI [25]. However, more research should be conducted in the future.

This study also suggested that the relationship between the BMI change observed in the study period (approximately 6 years) and the risk of MCI was partly affected by the baseline BMI. In subjects with a normal weight at baseline, an increased BMI in the study period was related to the onset of two types of MCI (aMCI and naMCI), but a decreased BMI in the same period was implicated only in the onset of aMCI. These findings were similar to the relationship observed between the baseline BMI and the risk of MCI.

In subjects who were overweight or obese at baseline, a further increase in BMI in the study period was not associated with an elevated risk of MCI, and a dose-effect relationship between BMI and MCI was not proved. In the same group of subjects, a decreased BMI in the study period showed a protective effect on aMCI risk, but this was not observed in subjects with naMCI. Furthermore, in subjects who were underweight at baseline, the study did not report an effect of increased BMI on MCI risk. The potential explanation was that the lack of data reduced the power of the test. More studies should thus be conducted.

There are several potential mechanisms that might explain the relationship between BMI and MCI risk. First, previous studies reported that overweight and obesity caused a variety of brain pathological changes, such as cerebral circulation insufficiency, neuronal injury and dysfunction, brain atrophy, inflammatory disorder, elevated β-amyloid precursor protein and tau protein expression [26–29], which might increase the MCI risk. Second, weight-loss-related energy dysmetabolism and hormone regulation disorder might affect the risk of the disease. Third, considering potential reverse causality, some prodromal symptoms of cognitive disorder (such as depression and apathy) could reduce appetite and cause weight loss [30]. Several dementia-related protein deposits have been documented in the olfactory pathway, and dysosmia may lead to appetite loss and maransis [31].

Previous studies have revealed that APOE4 might be an independent risk factor for AD and that APOE polymorphism might be involved in VD [32, 33]. Healthy people with APOE4 had a greater dementia risk, and dementia patients with APOE4 showed a worse response to therapy [34, 35]. In the study, APOE4 carriers were more common in the MCI groups than in the control group. Multivariate regression analysis, however, removed the effect of APOE polymorphism, and exciting results were reported.

## Conclusion

This study revealed that being overweight or obese may be an independent risk factor for aMCI and naMCI, as was weight gain in people who originally had a normal weight. Being underweight was another independent risk factor for aMCI but not for naMCI. Furthermore, weight loss in overweight/obese people and normal weight people might separately exert protective and pathogenic effects on aMCI. Taken together, overweight/obesity and weight gain might affect the risk of two types of MCI (aMCI and naMCI), and underweight and weight loss may only be implicated in the risk of aMCI. However, this is a preliminary study, so further research is necessary in large community based sample to validate our conclusion and reveal the mechanisms involved.

## Abbreviations

AD: Alzheimer’s disease
aMCI: Amnestic MCI
APOE4: Apolopoprotein E4
BMI: Body mass index
CHD: Coronary heart disease
CI: Confidence interval
DM: Diabetes mellitus
MCI: Mild cognitive impairment
MMSE: Mini-Mental State Examination
MoCA: Montreal Cognitive Assessment
MS: Metabolism syndrome
naMCI: Non-amnestic MCI
OR: Odds ratio
SD: Standard deviation
VD: Vascular dementia
